# Pandemic human-associated extended-spectrum β-lactamase-producing *Escherichia coli* lineages of ST38, ST131 and ST141 identified in Viennese dogs

**DOI:** 10.1093/jac/dkaf103

**Published:** 2025-04-01

**Authors:** Pia Saria, Pavlos G Doulidis, Amélie Desvars-Larrive, Adrienn Gréta Tóth, Iwan A Burgener, Alexandro Rodríguez-Rojas, Olga Makarova

**Affiliations:** Centre for Food Science and Veterinary Public Health, Clinical Department for Farm Animals and Food Systems Science, University of Veterinary Medicine, Vienna, Austria; Division for Small Animal Internal Medicine, Clinical Centre for Small Animals, Department for Small Animals and Horses, University of Veterinary Medicine, Vienna, Austria; Centre for Food Science and Veterinary Public Health, Clinical Department for Farm Animals and Food Systems Science, University of Veterinary Medicine, Vienna, Austria; Complexity Science Hub Vienna, Vienna, Austria; Centre for Bioinformatics, University of Veterinary Medicine, Budapest, Hungary; Department of Animal Breeding and Genetics, University of Veterinary Medicine, Budapest, Hungary; Division for Small Animal Internal Medicine, Clinical Centre for Small Animals, Department for Small Animals and Horses, University of Veterinary Medicine, Vienna, Austria; Division for Small Animal Internal Medicine, Clinical Centre for Small Animals, Department for Small Animals and Horses, University of Veterinary Medicine, Vienna, Austria; Centre for Food Science and Veterinary Public Health, Clinical Department for Farm Animals and Food Systems Science, University of Veterinary Medicine, Vienna, Austria

## Abstract

**Objectives:**

To assess the prevalence of ESBL Enterobacteriaceae among dogs attending a veterinary clinic in Vienna, characterize the isolates in terms of antimicrobial resistance, virulence and phylogenetic relationships.

**Methods:**

Faecal samples of 88 dogs were streaked on selective plates, species were identified by MALDI-ToF MS, tested for resistance by a combination disk test and VITEK 2^®^, whole genome-sequenced, bioinformatically genotyped, phylogenetically analysed and screened for resistance and virulence genes.

**Results:**

ESBL *Escherichia coli* carriage rate was 14.8% (95% CI: [8.1–23.9]). No carbapenem resistance was found, but 53.8% of the isolates were classified genotypically as multi-drug resistant. Phylogenetic analyses revealed that half of the isolates belonged to animal and environment-associated phylogroups, while another half was human-associated, and included high-risk international clones of ST38, ST131 and ST141, which clustered primarily with human isolates. All isolates harboured various virulence-associated genes, including four isolates that encoded exotoxins, of which two were from the pandemic ST131 and emerging ST141 lineages.

**Conclusions:**

Dogs in Vienna carry ESBL *E. coli* with high rates of multi-drug resistance and virulence, and a highly diverse population structure that includes pandemic human-associated lineages.

## Introduction

β-Lactam antibiotics are among the most widely used antimicrobial agents in the medical field. However, their efficacy is undermined by extended-spectrum β-lactamase (ESBL)-producing Enterobacteriaceae, which are typically resistant to penicillins, first-to-fourth generation cephalosporins and monobactams, but are susceptible to their combinations with β-lactam-inhibitors.^1^ ESBL-producing Enterobacteriaceae (ESBL-E) cause some of the most significant community- and hospital-associated infections, such as bloodstream, urinary and respiratory tract and wound infections.^2^ There is an increasing evidence that dogs can carry antimicrobial-resistant (AMR) bacteria,^3^ including ESBL-E.^4^ Considering that dogs are in frequent contact with humans, other animals and the environment, they may be an important but overlooked source of ESBL-E at the human–dog–environment interface. Little is known about the current rates of ESBL-E colonization among dogs in Austria: the most recent study conducted in Tyrol in 2012 found an ESBL *Escherichia coli* prevalence of 2.2% (*n* = 92).^5^ Therefore, the purpose of this study was to investigate the prevalence of ESBL-E in faecal samples of dogs attending a university veterinary hospital in Vienna, characterize these bacteria in terms of resistance to other antimicrobial agents, pathogenicity traits, clonal population structure and phylogenetic relationships.

## Materials and methods

### Ethics

All the samples used for this work were surplus materials from previous studies. The earlier studies were approved by the Ethics Committee of the University of Veterinary Medicine Vienna and the Austrian Federal Ministry of Science and Research (BMBWF-68.205/0216-V/3b/2019, 2021–0.518.574), and methods for sample collection were carried out by relevant Austrian guidelines and regulations.

### Sample collection

A total of 88 faecal samples from dogs visiting the Small Animal Clinic of the University of Veterinary Medicine in Vienna, Austria were collected between October 2022 and May 2023 using a convenience random sampling strategy. Patient information on the diagnosis and the possible link to an *E. coli* infection, in- or outpatient status, immunosuppression and antimicrobial treatment are available in Tables S1 and S2 (available as Supplementary data at *JAC* Online). Five grams of faeces was preserved in 500 μL DMSO, briefly frozen at −20°C, and then stored at −80°C until further processing.

### Bacterial isolation and antimicrobial susceptibility testing

Twenty-five microlitres of the faeces suspension was re-suspended in 225 μL LB medium (NutriSelect^®^, Sigma-Aldrich, USA), of which 20 µL was streaked on MacConkey Agar (Thermo Scientific^™^ Oxoid^™^, USA) plates supplemented with 2 μg/mL cefotaxime (Thermo Scientific^™^ Oxoid^™^, USA) and incubated for 24 h at 37°C. Single colonies of each morphology type were isolated, identified to a species level using MALDI-ToF MS (MALDI Biotyper^®^ (MBT), Bruker Corporation, USA) following the manufacturer’s instructions and stored at −80°C.

For ESBL-E screening, disk diffusion test was used according to EUCAST standards.^6^ The following disks were applied: cefotaxime (CTX) 30 µg, cefotaxime 30 µg + clavulanic acid (CTX/Clav) 10 µg and ceftazidime (CAZ) 30 µg. Additionally, a meropenem disk (MEM) 10 µg was applied to screen for carbapenem resistance. ESBL-E isolates were further tested by VITEK^®^ 2 (AES software, bioMérieux, Marcy l'Étoile, France) using VITEK^®^ 2 AST-N429 (bioMérieux) cards that contained the following antibiotics: piperacillin, piperacillin/tazobactam, cefotaxim, ceftazidime, cefepime, aztreonam, imipenem, meropenem, amikacin, gentamicin, tobramycin, ciprofloxacin, tigecycline, fosfomycin and trimethoprim/sulfamethoxazole. The strains *Klebsiella pneumoniae* ATCC 700603 (ESBL producer), *E. coli* ATCC 35218 (non-ESBL β-lactamase producer) and *E. coli* ATCC 25922 (susceptible non-ESBL-producing control strain) were used as quality control strains alongside the test strains. Susceptibility was interpreted according to EUCAST clinical breakpoints.^6^

### Whole genome sequencing and bioinformatic analyses

Illumina whole genome short-read sequencing (2 × 250 bp paired-end reads, 30× coverage) was performed by MicrobesNG (Birmingham, UK). Reference genomes were identified via Kraken, the reads were mapped using BWA mem and assembled using SPAdes, variant calling was performed using VarScan, and annotation was performed using Prokka.^7^ Multi-locus sequence typing (according to the Achtman MLST scheme^8^) and virulence-associated genes (VAGs) profiling (98% minimum identity, 60% minimum length) were performed in the CLC Genomics Workbench Version 23.0.5 (QIAGEN, Aarhus, Denmark). Antimicrobial resistance genes (ARGs) were identified with ResFinder-Version 4.6.0.,^9^ (90% identity, 100% minimum length). All ARGs found in the genomes were examined for whether the start and stop codons were the same as in the reference ARGs.

For phylogenetic analyses, short read datasets were downloaded from NCBI Sequence Read Archive (SRA) using the following keywords: ‘*Escherichia coli* ST38’, ‘*Escherichia coli* ST131’, ‘*Escherichia coli* ST141’ and ‘*Escherichia coli* AND dog OR canine OR canis among paired-end’, trimmed by Trimmomatic (v.0.39) (50 bp minimal length) and assembled by Spades (v4.0.0) (default settings). *Salmonella enterica*—GCF_000006945.2 was used to root the tree. All data manipulations were performed in R (v4.1.2). Phylogrouping was performed using Clermont typing scheme.^10^ Core genome neighbour-joining (cgNJ) trees and cladograms were constructed using ape based on Panaroo (v1.5.0) outputs created in strict modes with default settings using the ape package. MAFFT (v7.490) was used for multiple sequence alignment. Single nucleotide polymorphisms were identified using snp-sites (v2.5.). The best substitution model was selected by functions of phangorn (v2.11.1) package based on the Bayesian information criterion. Bootstrap values were produced by 100 iterations. Visualization was performed using the ggtree and ggtreeExtra packages in R. The complete list of references to the bioinformatic tools can be found in Table S3.

## Data availability

Sequence data have been deposited to the Nucleotide NCBI and SRA NCBI databases with the accession codes SAMN39585457–SAMN39585469 (Table S4) and the BioProject ID PRJNA1068331. The underlying code for this study is publicly available under https://github.com/tadrigreta/Ecoli_CODES_24.

## Results and discussion

### Antimicrobial resistance

**Figure 1.**
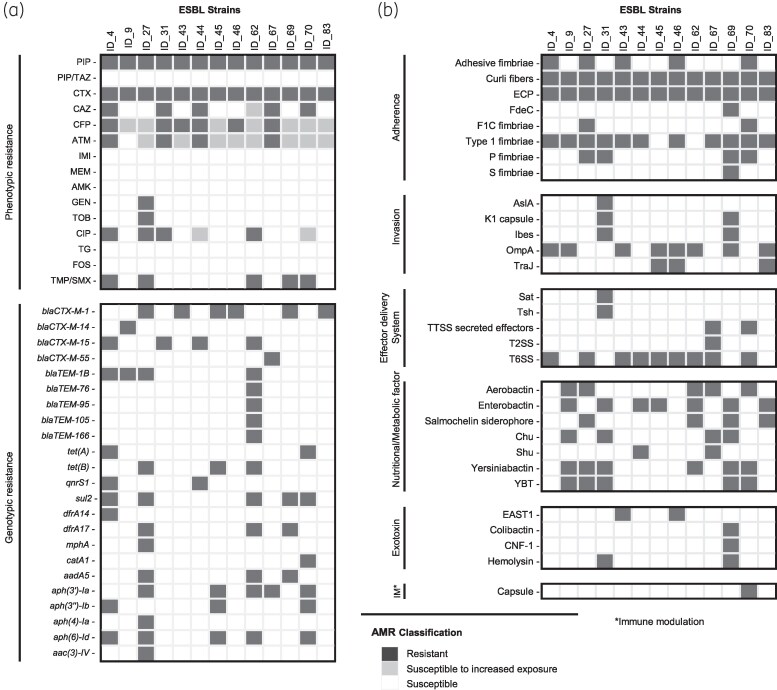
Genotypic and phenotypic resistance patterns observed in the ESBL isolates (a) and virulence-associated genes identified within the WGS of the ESBL isolates, grouped into functional categories (b). PIP, piperacillin; PIP/TAZ, piperacillin/tazobactam; CTX, cefotaxime; CAZ, ceftazidime; CFP, cefepime; ATM, aztreonam; IMI, imipenem; MEM, meropenem; AMK, amikacin; GEN, gentamicin; TOB, tobramycin; CIP, ciprofloxacin; TG, tigecycline; FOS, fosfomycin; TMP/SMX, trimethoprim/sulfamethoxazole.

Of the total 88 samples from dogs in Vienna, Austria, 13 were positive for ESBL *E. coli* (prevalence: 14.8%, 95% CI = [8.1–23.9]). This is considerably higher than the European average of 6.21% (95% CI: 3.24–11.58, *n* = 34 418)^11^ and approximately seven times higher than the 2.2% prevalence (*n* = 92) observed among dogs in Tyrol, Austria, in 2012.^5^ Phenotypic resistance to trimethoprim/sulfamethoxazole, tobramycin, gentamicin and ciprofloxacin was also present among the isolates. All tested isolates were susceptible to the carbapenem antibiotic meropenem, which is highly relevant to human medicine and is not approved in veterinary medicine.^12^ WGS revealed the presence of additional resistance genes and a multi-drug resistance (MDR) rate of 53.8%. The most prevalent β-lactamase-encoding genes were *bla*_CTX-M-1_ (6/13) and *bla*_CTX-M-15_ (4/13), which was in agreement with other studies of β-lactam ARGs in pets in Europe,^13^ as well as *bla*_TEM-1B_ (4/13). Other ARGs included *tet*(A), *dfrA14*, *qnrS1*, *mph*(A) and *aadA5*, conveying resistance to tetracycline, trimethoprim, quinolones, macrolides and streptomycin/spectinomycin, respectively (Figure 1(a), Tables S4–S6).

### Clonal structure and phylogenetic analyses

The phylogenetic analyses of 129 *E. coli* isolated primarily from dogs showed a wide distribution of the Viennese canine ESBL *E. coli* isolates across the phylogroups and geographies. The majority of the isolates from this study clustered within the clade with the predominantly European isolates, while only two isolates clustered within the North American clade. Over half of the isolates from this study belonged to the phylogroups associated with human intestinal (phylogroups C and A) and extraintestinal (phylogroups D, B2) *E. coli*, while four isolates belonged to the phylogroup B1, in which *E. coli* strains more prevalent in the environment and animals are typically found.^14^ MLST also revealed a diverse population structure with ESBL *E. coli* isolates belonging to 12 different sequence types: ST345, ST69, ST162, ST131, ST38, ST215, ST1079, ST4981, ST1011, ST141, ST88 and ST10 (Table 1, Figure S1).

**Table 1.** Phylogroups, ST, ARGs and plasmids identified in ESBL *E. coli* isolates

Bacterial strain ID	Accession Nr.	Clermont phylogroup	MLST	MDR^a^
ESBL_ID_4	SAMN39585457	B1	ST345	yes
ESBL_ID_9	SAMN39585458	D	ST69	no
ESBL_ID_27	SAMN39585459	B1	ST162	yes
ESBL_ID_31	SAMN39585460	B2	ST131	no
ESBL_ID_43	SAMN39585461	B1	ST1079	no
ESBL_ID_44	SAMN39585462	D1	ST38	no
ESBL_ID_45	SAMN39585463	A	ST215	yes
ESBL_ID_46	SAMN39585464	B1	ST1079	no
ESBL_ID_62	SAMN39585465	A	ST4981	yes
ESBL_ID_67	SAMN39585466	E	ST1011	no
ESBL_ID_69	SAMN39585467	B2	ST141	yes
ESBL_ID_70	SAMN39585468	C	ST88	yes
ESBL_ID_83	SAMN39585469	A	ST10	no

^a^Genotypic MDR, defined as resistance to three or more antimicrobial classes.

Strikingly, 23% (3/13) of isolates belonged to human-associated high-risk international pandemic extraintestinal pathogenic *E. coli* (ExPEC) clonal lineages ST38 and ST131 that often combine AMR and virulence^15,16^ and the emerging ExPEC clonal lineage ST141 associated with community-associated UTIs.^17^ The cgNJ trees for ST131, ST141 and ST38 containing WGS from humans, animals and the environment (Figure S2) revealed that the isolate ID_69 (ST141) clustered most closely with human clinical isolates from France and Poland, while being genetically highly divergent from them; the ID_44 isolate (ST38) was nested in the general cluster of predominantly avian samples from Northern Europe but was most closely related to two human clinical strains from the USA; while the isolate ID_31 (ST131) clustered with French and other European samples from both animals (mainly dogs and some cats) and humans (primarily from UTIs), but was genetically very distinct.

### Virulence traits

All ESBL-producing *E. coli* isolates harboured various virulence-associated genes, whereby genes encoding fimbriae, curli fibres and ECP (*E. coli* common pilus) that mediate adherence were the most common. Four isolates carried exotoxin-encoding VAGs: isolate ID_31 (pandemic ST131) harboured a haemolysin gene, isolates ID_43 and ID_46 from the animal-associated ST1079 carried the gene for the enteroaggregative *E. coli* heat-stable enterotoxin (EAST1 enterotoxin), and isolate ID_69 (emerging ST141) harboured genes for three exotoxins: haemolysin, cytotoxic necrotizing factor 1 and colibactin (Figure 1(b)).

Taken together, our data show that a considerable number of dogs in the greater Vienna metropolitan area carry ESBL-*E. coli*. Phylogenetic analyses suggest that despite the circulation of some clearly animal-associated lineages, dogs appear to be also colonized by *E. coli* from human-associated phylogroups that include high-risk international pandemic STs and encode exotoxins, suggesting that surveillance of AMR at the pet–human interface must adopt a One Health strategy.

## Supplementary Material

dkaf103_Supplementary_Data

## Supplementary data

Figures S1 and S2 and Tables S1–S6 are available as Supplementary data at *JAC* Online.
