# Correction to: Pandemic human-associated extended-spectrum β-lactamase-producing *Escherichia coli* lineages of ST38, ST131 and ST141 identified in Viennese dogs

**DOI:** 10.1093/jac/dkaf205

**Published:** 2025-06-27

This is a correction to: Pia Saria, Pavlos G Doulidis, Amélie Desvars-Larrive, Adrienn Gréta Tóth, Iwan A Burgener, Alexandro Rodríguez-Rojas, Olga Makarova, Pandemic human-associated extended-spectrum β-lactamase-producing *Escherichia coli* lineages of ST38, ST131 and ST141 identified in Viennese dogs, *Journal of Antimicrobial Chemotherapy*, Volume 80, Issue 6, June 2025, Pages 1573–1576, https://doi.org/10.1093/jac/dkaf103

In the originally published version of this manuscript, there was an error in the BioProject ID listed in the ‘Data availability’ section. The ID has been corrected from “PRJNA1069331” to “PRJNA1068331.”

