# Supplementary material for: Pandemic human-associated extended-spectrum β-lactamase-producing Escherichia coli lineages of ST38, ST131 and ST141 identified in Viennese dogs
Source: J Antimicrob Chemother. 2025 Apr 1;80(6):1573–6. doi: 10.1093/jac/dkaf103 (PMC12129576; doi:10.1093/jac/dkaf103)
Supplement: dkaf103_Supplementary_Data [file dkaf103_supplementary_data.docx]

**Supplementary material for the manuscript**

**“****Pandemic human-associated extended-spectrum β-Lactamase-producing *Escherichia coli* lineages from ST38, ST131 and ST141 identified in Viennese dogs”** by Pia SARIA^1^, Pavlos G. DOULIDIS^2^, Amélie DESVARS-LARRIVE^1,3^, Adrienn Gréta TÓTH^4,5^, Iwan A. BURGENER^2^, Alexandro RODRÍGUEZ-ROJAS^2^, Olga MAKAROVA^1^*

^1^Centre for Food Science and Veterinary Public Health, Clinical Department for Farm Animals and Food Systems Science, University of Veterinary Medicine, Vienna, Austria

^2^Division for Small Animal Internal Medicine, Clinical Centre for Small Animals, Department for Small Animals and Horses, University of Veterinary Medicine, Vienna, Austria

^3^Complexity Science Hub Vienna, Austria

^4^Centre for Bioinformatics, University of Veterinary Medicine, Budapest, Hungary

^5^Department of Animal Breeding and Genetics, University of Veterinary Medicine, Budapest, Hungary

*Correspondence: Olga Makarova
Telephone: +431250773303

E-mail: [olga.makarova@vetmeduni.ac.at](mailto:olga.makarova@vetmeduni.ac.at)

**Supplementary Figure S1.** Core-genome neighbour-joining cladogram representing 129 *E. coli* strains isolated primarily from dogs. The isolates from this study are highlighted.

**
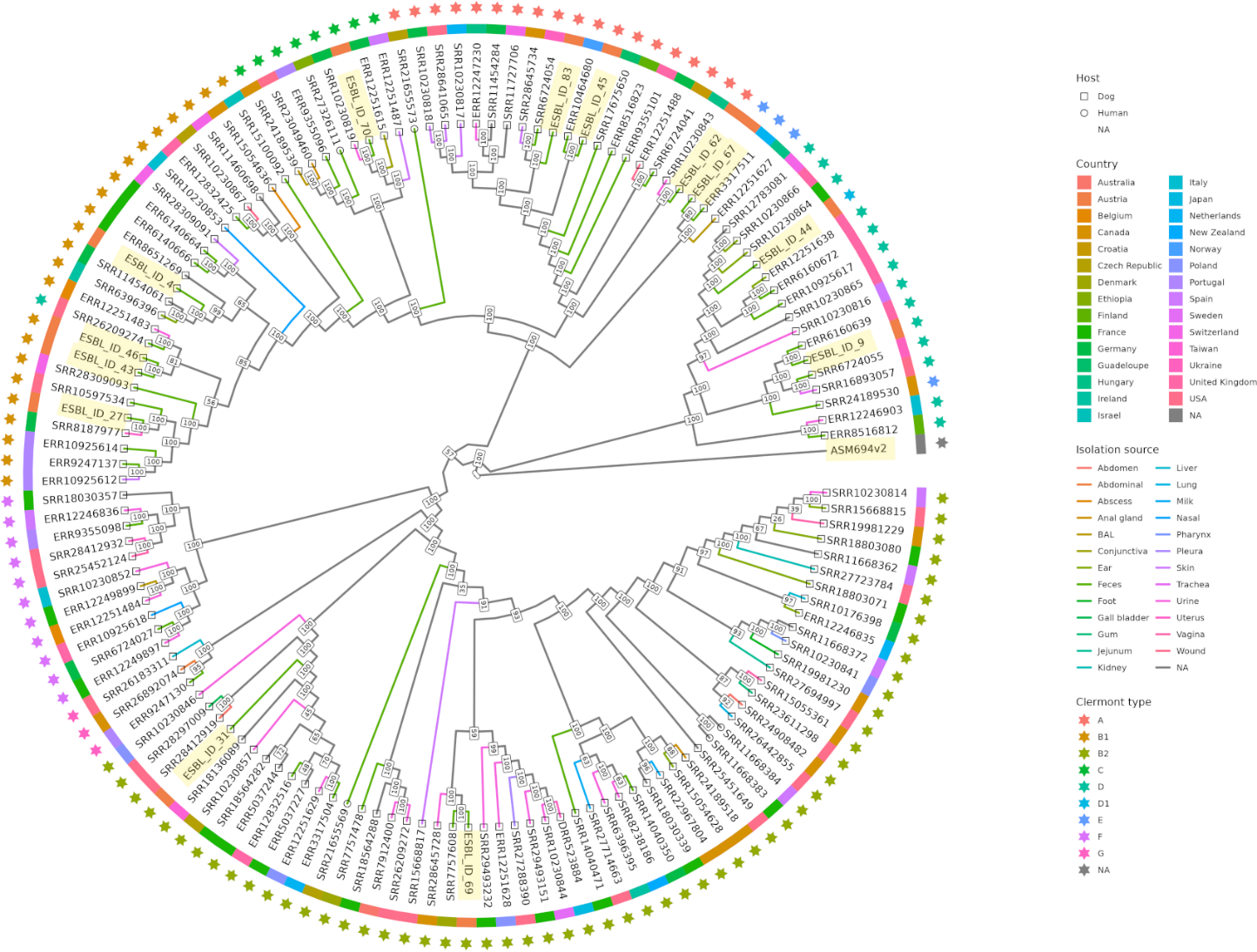
**

**Supplementary Figure S2**. Core-genome neighbour-joining trees and cladograms depicting the relationship between the strains from pandemic/emerging clones isolated in this study and other strains of the same ST type. (A) cgNJ tree of ESBL ID_69 among other ST141 isolates. (B) cgNJ tree of ESBL ID_44 among other ST38 isolates. (C) cgNJ cladogram of ID_31 among other ST131 isolates. The isolates from this study and the outgroup (*Salmonella enterica* GCF_000006945.2) are highlighted.


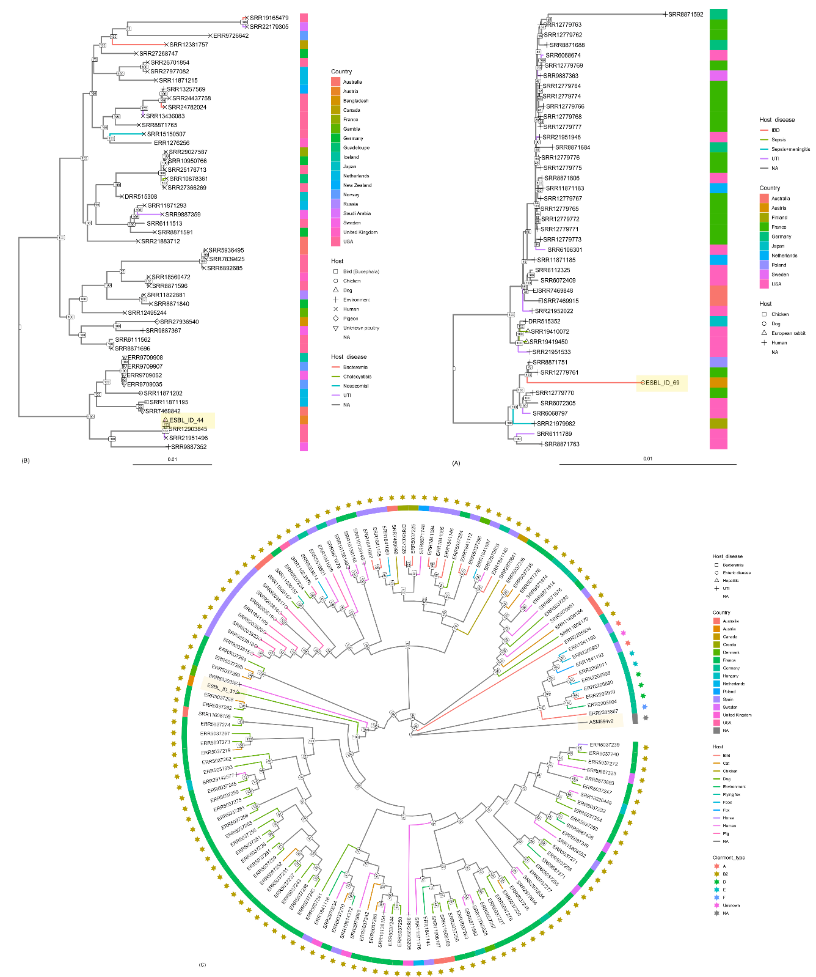


**Supplementary Table S1.** Information on the diagnoses of all dogs upon admission at the clinic investigated in this study.

| **Sample Number** | **Diagnosis** | **Immunosupression*** | **Antibiotics*** | **Antibiotics** |
| --- | --- | --- | --- | --- |
| 1 | bronchitis | yes | yes | quinolones |
| 2 | protein loss enteritis, sepsis | yes | yes | ⁠β-lactams, quinolones, tetracyclines |
| 3 | healthy | no | no |  |
| 4 | gastric dilation volvulus | no | no |  |
| 5 | gastrointestinal bleeding | no | no |  |
| 6 | acute leukemia | yes | yes | ⁠β-lactams, quinolones |
| 7 | diabetes mellitus | no | no |  |
| 8 | gastroenteritis | no | no |  |
| 9 | pyometra | no | yes | ⁠β-lactams, quinolones |
| 10 | healthy | no | no |  |
| 11 | healthy | no | no |  |
| 12 | pneumonia | no | yes | ⁠β-lactams, quinolones, tetracyclines, lincosamides |
| 13 | cerebellitis | yes | no |  |
| 14 | lymphoma | yes | yes | ⁠β-lactams |
| 15 | morbus addison | no | no |  |
| 16 | gastroenteritis | no | yes | ⁠β-lactams |
| 17 | acute hemorrhagic diarrhea syndrome | no | no |  |
| 18 | acute hemorrhagic diarrhea syndrome | no | no |  |
| 19 | gastroenteritis | no | no |  |
| 20 | post liver failure | yes | yes | ⁠β-lactams |
| 21 | healthy | no | no |  |
| 22 | acute hemorrhagic diarrhea syndrome | no | yes | ⁠β-lactams |
| 23 | healthy | no | no |  |
| 24 | acute hemorrhagic diarrhea syndrome | no | no |  |
| 25 | protein loss enteritis | yes | no |  |
| 26 | chronic enteropathy | no | no |  |
| 27 | orthopedic trauma | no | yes | ⁠β-lactams, quinolones, nitrofurantoin |
| 28 | canine atopic dermatitis | yes | no |  |
| 29 | healthy | yes | no |  |
| 30 | vasculitis | yes | yes | ⁠β-lactams |
| 31 | hepatitis | no | yes | ⁠β-lactams, quinolones |
| 32 | giardiosis | no | no |  |
| 33 | foreign body | no | no |  |
| 34 | epilepsy | no | no |  |
| 35 | gastroenteritis | no | yes | ⁠β-lactams, quinolones |
| 36 | acute hemorrhagic diarrhea syndrome | no | no |  |
| 37 | cholezystitis | no | yes | ⁠β-lactams |
| 38 | pankreatitis | no | yes | ⁠β-lactams |
| 39 | acute hemorrhagic diarrhea syndrome | no | no |  |
| 40 | acute hemorrhagic diarrhea syndrome | no | no |  |
| 41 | neoplasia in bladder | no | yes | ⁠β-lactams |
| 42 | ehrlichiosis | no | yes | ⁠β-lactams, tetracyclines |
| 43 | high-rise trauma | no | no |  |
| 44 | acute hemorrhagic diarrhea syndrome | no | yes | ⁠β-lactams, quinolones, macrolides |
| 45 | healthy | no | no |  |
| 46 | acute hemorrhagic diarrhea syndrome | no | yes | ⁠β-lactams |
| 47 | acute hemorrhagic diarrhea syndrome | no | yes | ⁠β-lactams |
| 48 | acute hemorrhagic diarrhea syndrome | no | yes | ⁠β-lactams |
| 49 | protein loss enteritis | no | no |  |
| 50 | morbus addison | no | no |  |
| 51 | chronic enteropathy | no | no |  |
| 52 | intoxication | no | no |  |
| 53 | acute hemorrhagic diarrhea syndrome | no | no |  |
| 54 | histiocytic colitis | no | yes | quinolones |
| 55 | chronic enteropathy | no | no |  |
| 56 | immune-mediated hemolytic anemia | yes | no |  |
| 57 | acute hemorrhagic diarrhea syndrome | no | no |  |
| 58 | healthy | no | no |  |
| 59 | gastroenteritis | no | yes | ⁠β-lactams |
| 60 | chronic kidney disease | no | yes | ⁠β-lactams, quinolones |
| 61 | gastroenteritis | no | yes | ⁠β-lactams |
| 62 | pankreatitis | no | yes | quinolones |
| 63 | ahds | no | yes | ⁠β-lactams |
| 64 | gastritis | no | no |  |
| 65 | acute hemorrhagic diarrhea syndrome | no | no |  |
| 66 | morbus addison | no | no |  |
| 67 | gastric ulcer | no | yes | ⁠β-lactams |
| 68 | nystagmus | no | no |  |
| 69 | inflammatory bowel disease | no | no |  |
| 70 | lymphoma | no | yes | ⁠β-lactams, quinolones |
| 71 | acute hemorrhagic diarrhea syndrome | no | yes | ⁠β-lactams |
| 72 | inflammatory bowel disease | no | no |  |
| 73 | liver carcinoma | no | no |  |
| 74 | chronic enteropathy | no | no |  |
| 75 | acute hemorrhagic diarrhea syndrome | no | no |  |
| 76 | chronic enteropathy | no | no |  |
| 77 | acute hemorrhagic diarrhea syndrome | no | yes | ⁠β-lactams, quinolones |
| 78 | blepharitis | yes | no |  |
| 79 | ileus | no | no |  |
| 80 | acute hemorrhagic diarrhea syndrome | no | yes | ⁠β-lactams |
| 81 | excluded from analysis** |  |  |  |
| 82 | healthy | no | no |  |
| 83 | inflammatory bowel disease | no | no |  |
| 84 | hypercalcemia | no | no |  |
| 85 | acute hemorrhagic diarrhea syndrome | no | no |  |
| 86 | pankreatitis | no | no | nitroimidazole |
| 87 | healthy | no | yes | ⁠β-lactams |
| 88 | healthy | no | no |  |
| 89 | healthy | no | no |  |

*received in 6 months before sample was taken

**sample was excluded from analysis due to missing data

**Supplementary Table S2.** Information on the diagnoses of the dogs upon admission at the clinic investigated that were tested ESBL-positive.

| **Sample Number** | **Diagnosis** | **Possible Link to *E. coli*** | **Inpatient around time of sampling** |
| --- | --- | --- | --- |
| 4 | Gastric Dilation Volvulus | no | yes |
| 9 | Pyometra | yes | yes |
| 27 | Orthopedic implant infection | no | yes |
| 31 | Hepatitis | no | yes |
| 43 | High Rise Trauma | no | yes |
| 44 | AHDS (Acute Hemorrhagic Diarrhea Syndrome) | yes | yes |
| 45 | healthy | no | no |
| 46 | AHDS | yes | yes |
| 62 | Pankreatitis | no | yes |
| 67 | Ulcer | no | yes |
| 69 | IBD (Inflammatory Bowel Disease) | yes | no |
| 70 | Lymphoma | no | yes |
| 81 | Hepatitis | no | yes |
| 83 | IBD (Inflammatory Bowel Disease) | yes | no |

**Supplementary Table S3.** References to the bioinformatic tools used for phylogenetic and population structure analysis

| **Tool** | **Reference** |
| --- | --- |
| SRA | Leinonen R, Sugawara H, Shumway M. The Sequence Read Archive. *Nucleic Acids Res* 2011; 39(Database issue): D19–21. |
| R | R Core Team. R: A language and environment for statistical computing. <http://www.r-project.org>. |
| REFSEQ | O'Leary NA, Wright MW, Brister JR et al. Reference Sequence (RefSeq) database at NCBI: current status, taxonomic expansion, and functional annotation. Nucleic Acids Res 2016; 44: D733–45. |
| TRIMMOMATIC | Bolger AM, Lohse M, Usadel B. Trimmomatic: A flexible trimmer for Illumina sequence data. *Bioinformatics* 2014; 30: 2114–20. |
| SPADES | Prjibelski A, Antipov D, Meleshko D et a l. Using SPAdes de novo assembler. *Curr Protoc Bioinformatics* 2020; 70(1): e102. |
| CLERMONTYPING | Beghain J, Bridier-Nahmias A, Le Nagard H et al. ClermontTyping: An easy-to-use and accurate in silico method for Escherichia genus strain phylotyping. Microb Genom 2018; 4: e000192. |
| APE | Paradis E, Schliep K. APE 5.0: An environment for modern phylogenetics and evolutionary analyses in R. Bioinformatics 2019; 35: 526–8. |
| PANAROO | Tonkin-Hill G, MacAlasdair N, Ruis C et al. Producing polished prokaryotic pangenomes with the Panaroo pipeline. Genome Biol 2020; 21: 180. |
| MAFFT | Katoh K, Misawa K, Kuma K et al . MAFFT: A novel method for rapid multiple sequence alignment based on fast Fourier transform. Nucleic Acids Res 2002; 30: 3059–66. |
| SNP-SITES | Page AJ, Taylor B, Delaney AJ et al . SNP-sites: Rapid efficient extraction of SNPs from multi-FASTA alignments. Microbial Genomics 2016; 2: e000056. |
| PHANGORN | Schliep KP. Phangorn: Phylogenetic analysis in R. Bioinformatics 2011; 27: 592–3. |
| GGTREEEXTRA | Xu S, Dai Z, Guo P et al. ggtreeExtra: Compact visualization of richly annotated phylogenetic data. Mol Biol Evol 2021; 38: 4039–42. |
| GGTREE | Yu G. Using ggtree to visualize data on tree-like structures. Current Protocols in Bioinformatics 2020; 69: E96. |

­­ **Supplementary Table S4.** Additional information on the isolates obtained by WGS. The assembled sequences were uploaded to Nucleotide NCBI database and the short-reads to SRA NCBI under the given accession numbers. Species was determined via 16S analysis. The assembly size and GC content was provided by MicrobesNG. The Number of ARGs were determined ResFinder-4.6.0 with 90% threshold for Identification and a 100% threshold for minimum length, all ARGs found in the genomes were had the same start and stop codons as in the reference ARGs.

| **Faecal Sample** | | **Bacterial Strain ID** | | **Accession Nr.** | **Species** | **Assembly size** | **GC content** | **ARGs** |
| --- | --- | --- | --- | --- | --- | --- | --- | --- |
| **4** | ID_4 | | SAMN39585457 | | *E.coli* | 4652889 bp | 50.64 % | *aph(6)-Id, aph(3'')-Ib, bla*_CTX-M-15_*, bla*_TEM-1B_*, qnrS1, sul2, tet*(A)*, dfrA14* |
| **9** | ID_9 | | SAMN39585458 | | *E.coli* | 4670941 bp | 50.44 % | *bla*_TEM-1B_*, bla*_CTX-M-14_ |
| **27** | ID_27 | | SAMN39585459 | | *E.coli* | 4672659 bp | 50.42 % | *aph(6)-Id, aph(3'')-Ib, aph(4)-Ia, aac(3)-IV, aadA5, aph(3')-Ia, bla*_TEM-1B_*, bla*CTX-M-1*, mph*(A)*, sul2, tet*(B)*, dfrA17* |
| **31** | ID_31 | | SAMN39585460 | | *E.coli* | 4673035 bp | 50.42 % | *bla*_CTX-M-15_ |
| **43** | ID_43 | | SAMN39585461 | | *E.coli* | 4974724 bp | 50.71 % | *bla*_CTX-M-1_ |
| **44** | ID_44 | | SAMN39585462 | | *E.coli* | 4665379 bp | 50.5 % | *bla*_CTX-M-15_*, qnrS1* |
| **45** | ID_45 | | SAMN39585463 | | *E.coli* | 4712079 bp | 50.69 % | *aph(6)-Id, aph(3')-Ia, aph(3'')-Ib, bla*_CTX-M-1_*, tet*(B) |
| **46** | ID_46 | | SAMN39585464 | | *E.coli* | 4666637 bp | 50.49 % | *bla*_CTX-M-1_ |
| **62** | ID_62 | | SAMN39585465 | | *E.coli* | 4666855 bp | 50.47 % | *aph(6)-Id, aph(3'')-Ib, aadA5, aph(3')-Ia, bla*_TEM-166_*, bla*_TEM-105_*, bla*_TEM-95_*, bla*_TEM-76_*, bla*_TEM-1B_*, bla*_CTX-M-15_*, sul2, tet*(B), *dfrA17* |
| **67** | ID_67 | | SAMN39585466 | | *E.coli* | 4677851 bp | 50.37 % | *aph(3')-Ia, bla*_CTX-M-55_ |
| **69** | ID_69 | | SAMN39585467 | | *E.coli* | 4676459 bp | 50.39 % | *bla*_CTX-M-1_*, aadA5, sul2, dfrA17* |
| **70** | ID_70 | | SAMN39585468 | | *E.coli* | 4673111 bp | 50.42 % | *aph(6)-Id, aph(3'')-Ib, aph(3')-Ia, catA1, sul2, tet*(A) |
| **83** | ID_83 | | SAMN39585469 | | *E.coli* | 4661485 bp | 50.53 % | *bla*_CTX-M-1_ |

­

**Supplementary Table S5.** Disc Diffusion results and interpretations according to EUCAST clinical breakpoints (2023) Isolates resistant/intermediate to one or both cephalosporins were positive for ESBL if the increase in zone diameter in the presence of clavulanic acid was ≥ 5 mm. (EUCAST guidelines for detection of resistance mechanism and specifi specific resistances of clinical and/or epidemiological importance: Version 2.0). CTX = cefotaxime, CAZ = ceftazidime, CTX/CLAV = cefotaxime/clavulanic acid, MEM = meropenem, R = resistant, I = susceptible to increased exposure, S = susceptible.

| Faecal Sample | Bacterial Strain ID | CTX (mm) | CTX | CAZ (mm) | CAZ | CTX/CLAV (mm) | MEM (mm) | MEM | ESBL |
| --- | --- | --- | --- | --- | --- | --- | --- | --- | --- |
| **4** | ID_4 | 12 | R | 23 | S | 33 | 34 | S | yes |
| **9** | ID_9 | 16 | R | 27 | S | 29 | 32 | S | yes |
| **27** | ID_27 | 15 | R | 31 | S | 32 | 36 | S | yes |
| **31** | ID_31 | 14 | R | 22 | S | 32 | 33 | S | yes |
| **43** | ID_43 | 18 | I | 19 | I | 31 | 30 | S | yes |
| **44** | ID_44 | 10 | R | 23 | S | 31 | 32 | S | yes |
| **45** | ID_45 | 11 | R | 20 | I | 19 | 31 | S | yes |
| **46** | ID_46 | 19 | I | 29 | S | 32 | 30 | S | yes |
| **62** | ID_62 | 18 | I | 21 | I | 30 | 33 | S | yes |
| **67** | ID_67 | 12 | R | 20 | I | 29 | 31 | S | yes |
| **69** | ID_69 | 11 | R | 29 | S | 32 | 34 | S | yes |
| **70** | ID_70 | 19 | I | 18 | R | 24 | 34 | S | yes |
| **83** | ID_83 | 17 | R | 28 | S | 32 | 34 | S | yes |

**Supplementary Table S6.** Phenotypic antimicrobial resistance determined via VITEK2 using the AST-N429 and interpreted according to EUCAST 2024 clinical breakpoints. PIP = piperacillin, PIP/TAZ = piperacillin/tazobactam, CTX = cefotaxime, CAZ = ceftazidime, CFP= cefepime, ATM = aztreonam, IMI = imipenem, MEM = meropenem, AMK = amikacin, GEN = gentamycin, TOB = tobramycin, CIP = ciprofloxacin, TG = tigecycline, FOS = Fosfomycin, TMP/SMX = trimethoprim/sulfamethoxazole, R = resistant, I = susceptible to increased exposure, S = susceptible.

| Faecal Sample | Bacterial Strain ID | Species | Phenotype | MDR^1^ | | PIP | | PIP/ TAZ | | CTX | | CAZ | | CFP | | ATM | | IMI | MEM | AMK | GEN | TOB | CIP | TG | FOS | | TMP/SMX | |
| --- | --- | --- | --- | --- | --- | --- | --- | --- | --- | --- | --- | --- | --- | --- | --- | --- | --- | --- | --- | --- | --- | --- | --- | --- | --- | --- | --- | --- |
| 4 | ID_4 | *E. coli* | ESBL | yes | R | | S | | R | | R | | R | | R | | S | | S | S | S | S | R | S | S | R | |  |
| 9 | ID_9 | *E. coli* | ESBL | no | R | | S | | R | | S | | I | | S | | S | | S | S | S | S | S | S | S | S | |  |
| 27 | ID_27 | *E. coli* | ESBL | yes | R | | S | | R | | S | | I | | I | | S | | S | S | R | R | R | S | S | R | |  |
| 31 | ID_31 | *E. coli* | ESBL | no | R | | S | | R | | R | | R | | R | | S | | S | S | S | S | R | S | S | S | |  |
| 43 | ID_43 | *E. coli* | ESBL | no | R | | S | | R | | S | | R | | I | | S | | S | S | S | S | S | S | S | S | |  |
| 44 | ID_44 | *E. coli* | ESBL | no | R | | S | | R | | R | | R | | R | | S | | S | S | S | S | I | S | S | S | |  |
| 45 | ID_45 | *E. coli* | ESBL | no | R | | S | | R | | S | | I | | I | | S | | S | S | S | S | S | S | S | S | |  |
| 46 | ID_46 | *E. coli* | ESBL | no | R | | S | | R | | S | | R | | I | | S | | S | S | S | S | S | S | S | S | |  |
| 62 | ID_62 | *E. coli* | ESBL | yes | R | | S | | R | | I | | I | | I | | S | | S | S | S | S | R | S | S | R | |  |
| 67 | ID_67 | *E. coli* | ESBL | no | R | | S | | R | | R | | R | | R | | S | | S | S | S | S | S | S | S | S | |  |
| 69 | ID_69 | *E. coli* | ESBL | no | R | | S | | R | | S | | I | | I | | S | | S | S | S | S | S | S | S | R | |  |
| 70 | ID_70 | *E. coli* | ESBL | no | R | | S | | R | | R | | I | | I | | S | | S | S | S | S | I | S | S | R | |  |
| 83 | ID_83 | *E. coli* | ESBL | no | R | | S | | R | | S | | I | | I | | S | | S | S | S | S | S | S | S | S | |  |

^1^phenotypic MDR according to VITEK panel only
